# Bradycardia, Renal Failure, Atrioventricular (AV) Nodal Blockers, Shock, and Hyperkalemia (BRASH) Syndrome: A Confounder in the Clinical Practice

**DOI:** 10.7759/cureus.72793

**Published:** 2024-10-31

**Authors:** Mariana Certal, Diana Mimoso, Beatriz R Marques, Elisabete Cerqueira, Beatriz Exposito

**Affiliations:** 1 Internal Medicine, Unidade Local de Saúde de Trás-os-Montes e Alto Douro, Chaves, PRT; 2 Internal Medicine, Centro Hospitalar de Trás-os-Montes e Alto Douro, Chaves, PRT

**Keywords:** atrioventricular nodal block, brash syndrome, hyperkalemia management, renal insufficiency, severe bradycardia

## Abstract

BRASH, an acronym for Bradycardia, Renal failure, AV nodal blockers, Shock, and Hyperkalemia, syndrome is a clinical synergic phenomenon that can result in cardiovascular collapse. We present the case of an 83-year-old woman with dilated cardiomyopathy, heart failure, and chronic kidney disease who was admitted to the emergency room due to syncope and a worsening general condition. The patient was on several medications, including carvedilol, a beta-blocker. On physical examination, she was found to be hypotensive and bradycardic, with no other significant findings. Laboratory results revealed urea of 161 mg/dL, creatinine of 2.7 mg/dL, and potassium of 5.2 mEq/L. The electrocardiogram showed bradycardia with a regular junctional rhythm without signs of ischemia or other alterations. Given the patient's bradycardia, hyperkalemia, and renal dysfunction, atropine was administered, but no significant clinical response was observed. The patient’s condition worsened, with progressive bradycardia, oliguria, and neurological dysfunction. Based on the combination of these findings, a diagnosis of BRASH syndrome was made. Treatment was initiated with isoproterenol to stimulate heart rate, along with fluid therapy, calcium gluconate, and insulin to address hyperkalemia. The bradycardia gradually improved, allowing the discontinuation of isoproterenol after 24 hours.

The BRASH syndrome is a potentially life-threatening condition that can go unrecognized without early identification. This case underscores the importance of swift diagnosis and timely intervention in managing BRASH syndrome. The combination of factors, including hyperkalemia, bradycardia, and renal failure in patients on atrioventricular nodal blockers, should always raise suspicion for this condition. Rapid and targeted therapy is essential to prevent adverse outcomes and ensure the patient’s recovery.

## Introduction

Bradycardia, Renal Insufficiency, Atrioventricular (AV) Nodal Block, Shock, and Hyperkalemia (BRASH) Syndrome was first described in 2016 and refers to a set of signs resulting from the synergistic effect of atrioventricular (AV) nodal blocking medications and renal insufficiency, which can lead to hemodynamic and cardiovascular collapse [1].

The pathophysiology of this syndrome primarily results from the interplay between AV nodal block and hyperkalemia, resulting in a vicious cycle that perpetuates and exacerbates each disorder [1]. An apparently innocuous event, such as initial dehydration, can slightly reduce renal perfusion and the glomerular filtration rate (GFR), causing the accumulation of drugs eliminated by the kidneys, such as beta-blockers, non-dihydropyridine calcium channel blockers, and other antiarrhythmics [2,3]. Their continuous accumulation results in bradycardia, decreased cardiac output, and consequently, progression of renal insufficiency. On the other hand, renal injury can result in a decreased glomerular filtration rate, impeding potassium excretion, enhancing its reabsorption in the kidneys, disrupting aldosterone production, and leading to metabolic acidosis, which worsens potassium accumulation in the blood. This condition also diminishes the excretion of medications that rely on renal function for their elimination. Thus, although therapeutic doses of these drugs generally do not cause severe bradycardia, reduced renal clearance and concomitant hyperkalemia intensify the effects of AV nodal blockers [2,3], developing a cycle that can progress to shock and subsequent multi-organ failure [4].

This syndrome is particularly relevant in elderly patients with cardiac pathology and renal disease who are treated with AV nodal-blocking medications, such as β-blockers or non-dihydropyridine calcium channel blockers, notably verapamil or diltiazem [2]. Hypovolemia has been recognized as the most common precipitating factor, although other triggers have been identified, including increasing doses of antihypertensive medications and the combination or increased doses of inhibitors of the renin-angiotensin-aldosterone system [3].

Although the isolated effects of the different entities have been widely described [5], the recognition and characterization of BRASH syndrome as a distinct entity is relatively recent. Some of the characteristics that distinguish this syndrome include the fact that patients typically present with only mild to moderate hyperkalemia, contrary to studies showing that only severe hyperkalemia (for example, potassium > 7 mEq/L) causes bradycardia [6]. Moreover, the electrocardiogram (ECG) of patients usually shows severe bradycardia without other electrocardiographic features of hyperkalemia, such as peaked T waves [3,7].

The treatment of BRASH syndrome involves hemodynamic support, immediate correction of hyperkalemia, and treating bradycardia. There is a need for a more aggressive correction of hyperkalemia, even if mild, with intravenous administration of insulin, glucose, and calcium for membrane stabilization (typically used only in severe hyperkalemia). The BRASH syndrome is a clinical entity with a variable presentation that, despite being emerging, is underdiagnosed and often confused with less complex abnormalities. Therefore, a detailed understanding of the underlying pathophysiological mechanism is essential to optimize treatment strategies and prevent progression to serious complications.

## Case presentation

An 83-year-old female patient with a history of dilated cardiomyopathy, New York Heart Association (NYHA) class III heart failure with a left ventricular ejection fraction of 46%, chronic kidney disease stage G3a, hypertension, dyslipidemia, and type 2 diabetes mellitus was admitted to the emergency department for syncope. She denied respiratory, gastrointestinal, or genitourinary symptoms. Six days prior, she had been evaluated in the same department for worsening general condition, without other complaints. She was diagnosed with a urinary tract infection and treated with ciprofloxacin. At the time of observation, she was taking carvedilol, olmesartan, amlodipine, hydrochlorothiazide, furosemide, pravastatin, dapagliflozin, quetiapine, and trazodone, with no recent changes to her medication regimen.

On examination, she was conscious and oriented, with no neurological deficits, no signs of respiratory distress, afebrile, hypotensive (blood pressure 96/56 mmHg), and bradycardic (heart rate 41 beats per minute (bpm)). Laboratory results revealed a blood urea level of 161 mg/dL, creatinine of 2.7 mg/dL, potassium (K+) of 5.2 mEq/L, sodium (Na+) of 134 mEq/L, C-reactive protein (CRP) of 2.14 mg/dL, troponin T of 0.047 ng/mL, myoglobin of 66.4 ng/mL, pro-B-type natriuretic peptide (BNP) of 2288 pg/mL, lactate of 0.7 mmol/L, and bicarbonate of 17.5 mEq/L. There was no evidence of hepatic cytolysis, hyperbilirubinemia, or thyroid function abnormalities (Table 1).

**Table 1:** Analytical study at admission ALT: Alanine transaminase; aPTT: Activated partial thromboplastin time; AST: Aspartate transaminase; CRP: C-reactive protein, INR: International normalized ratio; LDH: Lactate dehydrogenase; pCO2: Partial pressure of carbon dioxide, pO2: Partial pressure of oxygen; TB: Total bilirubin; γ-GT: Gamma-glutamyl transferase

Parameter	Result	Reference range
Hemoglobin	10.5	12 - 16 g/dL
Leukocytes	6910	4000 - 11000/µL
Platelets	191000	150000 - 400000/µL
CRP	2.14	< 0.5 mg/L
Urea	161	0 - 50 mg/dL
Creatinine	2.7	0.6 - 1.1 mg/dL
Sodium	134	135 - 147 mEq/L
Potassium	5.2	3.7 - 5.1 mEq/L
Chlorides	103	96 - 106 mEq/L
Total calcium	9.0	8,8 - 10.2 mg/dL
Magnesium	2.7	1.58 - 2.55 mg/dL
AST	12	< 35 U/L
ALT	7.0	< 33 U/L
γ-GT	9.0	7 - 32 U/L
LDH	102	135 - 214 U/L
TB	0.2	< 1.2 mg/dL
Troponin T	0.047	< 0.05 ng/mL
Myoglobin	66.4	< 58 ng/mL
Pro-BNP	2288	< 120 pg/mL
INR	1.18	<1.2
aPTT	30,7	27 - 38 seconds
pH	7,42	7,35 - 7,45
pO_2_	71	80 - 100 mmHg
pCO_2_	27	35 - 45 mmHg
Lactate	0,7	0,63 - 2,44 mmol/L
Bicarbonate	17,5	22 - 26 mEq/L

**Figure 1:**
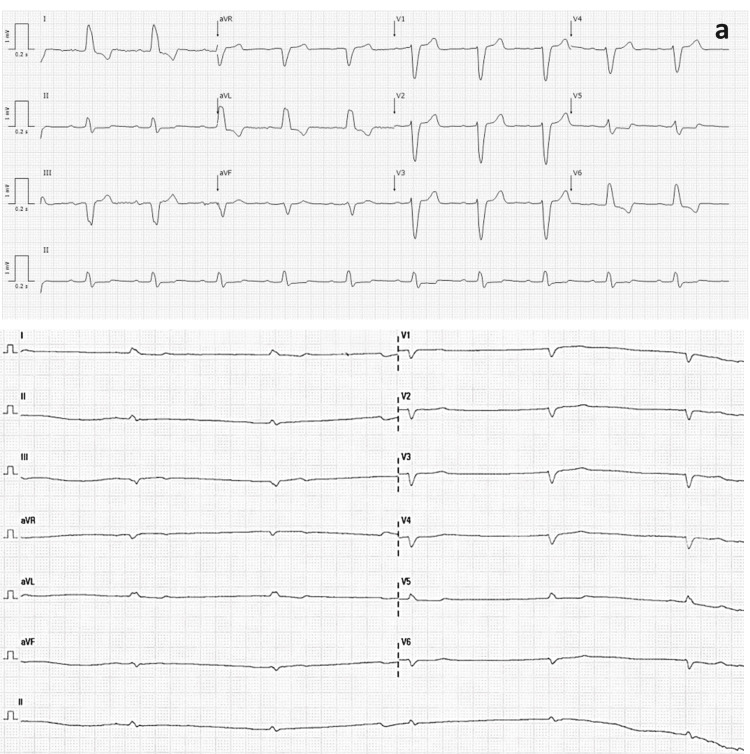
Previous electrocardiogram of the patient Previous electrocardiogram showing sinus bradycardia (heart rate (HR) 55 bpm) (a) and the electrocardiogram at the time of admission showing bradycardia with a regular junctional rhythm, without signs of ischemia or other alterations (HR 32 bpm) (b).

The urinalysis showed leukocyturia, and the urine culture identified an ESBL (extended-spectrum beta-lactamase) *Klebsiella pneumoniae*. The ECG displayed bradycardia with a regular junctional rhythm, without signs of ischemia or other alterations (Figure 1).

The chest X-ray showed no significant abnormalities. Atropine (0.5 mg) was administered twice, with no response. The patient progressed with worsening hypotension (BP 78/43 mmHg) and bradycardia (HR 30-40 bpm), along with oliguria and neurological dysfunction. A diagnosis of BRASH syndrome was made. Treatment was initiated with a titrated isoproterenol infusion to achieve a heart rate > 60 bpm, fluid therapy, intravenous calcium gluconate, 10 units of regular insulin in 10% dextrose, which was repeated twice, and meropenem due to suspected urinary tract infection in a patient with multiple prior courses of antibiotic therapy and isolation of multidrug-resistant microorganisms in the urine. The bradycardia gradually resolved, allowing for the discontinuation of isoproterenol 24 hours later, coinciding with improvements in renal function and hyperkalemia. The patient was hospitalized for eight days and was discharged with a stabilized heart rate and without changes in renal function. The patient was referred for outpatient follow-up.

## Discussion

The BRASH syndrome is characterized by the synergy between mild hyperkalemia and therapeutic levels of AV node blockers, resulting in a vicious cycle that can progress to shock and multiple organ failure [1]. This syndrome is triggered by drug-metabolic interactions and is more prevalent in the elderly population with cardiac and renal pathology, usually on multiple medications. Thus, in the presence of bradycardia, hyperkalemia, and a pharmacological history of AV node blockers, this diagnosis should always be considered to facilitate the implementation of a rapid and effective therapeutic intervention.

In the presented case, the suspicion of BRASH syndrome was based on the identification of characteristic clinical signs and the exclusion of alternative diagnoses, such as sepsis, acute cardiac disease, or decompensated hypothyroidism, since the patient had normal levels of thyroid-stimulating hormone (TSH) and free T4. The patient had a history of chronic kidney disease stage G3a, and in her pharmacological history, treatment with carvedilol 6.25 mg administered twice daily was highlighted, as prescribed by her physician, with no evidence of overdose. In the various reported cases of BRASH syndrome, patients adhered correctly to the dosage of medications and rarely presented supratherapeutic levels. However, β-blocker overdose constitutes a differential diagnosis in this context [3]. The BRASH syndrome can have a highly variable presentation, and a common error leading to underdiagnosis is the focus on a single aspect of the syndrome instead of considering all the factors involved in its pathogenesis.

The vicious cycle is generally initiated by hypovolemia or by medications that block the AV node, and if not diagnosed quickly, it can progress to shock [3]. In the presented case, the β-blocking effect of carvedilol may have aggravated the hyperkalemia and the spiral of events. On the other hand, hyperkalemia, in this context, also constituted an important factor in the development of bradyarrhythmia, despite the ECG showing bradycardia without the classic signs of hyperkalemia, such as peaked T waves [7], as previously described in this syndrome [3,7].

The rapid establishment of hemodynamic support, correction of hyperkalemia, and discontinuation of the β-blocker were critical factors for the patient's recovery, without the need for renal replacement therapy or pacemaker placement.

In BRASH syndrome, the treatment goals are (1) to correct hyperkalemia, (2) to provide hemodynamic support for bradycardia and hypotension, and (3) to treat the triggering events (e.g., hypovolemia). Early administration of intravenous calcium stabilizes the cardiac membrane and improves cardiac output, although it does not reduce the extracellular concentration of potassium [8]. To promote the transport of potassium into the cells, intravenous insulin and dextrose are used, as well as β-agonists, although the latter are less effective when administered via inhalation. To reduce total body potassium, sodium polystyrene sulfonate is often used, which binds potassium in the colon in exchange for sodium, promoting its excretion [8]. Sodium zirconium cyclosilicate has a similar mechanism but with a shorter duration of action [4,8]. Identifying and correcting the triggering cause is also essential. Hypovolemic patients should receive Ringer's lactate solution or isotonic bicarbonate, especially if there is acidosis. Excessive administration of normal saline solution can induce hyperchloremic metabolic acidosis, worsening hyperkalemia by displacing potassium into the extracellular space. However, volume resuscitation must be closely monitored to avoid volume overload, which can further compromise hemodynamic status.

In the presented case, in addition to the discontinuation of the β-blocker and the institution of fluid therapy and isoproterenol infusion, it was essential to adopt aggressive treatment for hyperkalemia. The administration of membrane-stabilizing drugs, such as intravenous calcium and insulin, should be considered even for mild hyperkalemia, aiming to promote a more effective correction of the electrolyte disturbance.

This case highlights the importance of considering BRASH syndrome as a clinical entity in at-risk populations, namely polymedicated elderly patients, especially in light of the synergistic combination of bradycardia, renal insufficiency, the use of renin-angiotensin system blockers, and hyperkalemia. Understanding the underlying pathophysiology is essential for a more appropriate clinical approach aimed at preventing potentially fatal hemodynamic complications.

Given the progressive aging of the population and the increasingly stringent blood pressure therapeutic targets in the treatment of hypertension (HTA), BRASH syndrome emerges as a condition with a growing potential for incidence in clinical practice. Therefore, it becomes imperative that healthcare professionals are capable of early identification of associated signs and symptoms in order to optimize therapeutic interventions and reduce morbidity and mortality related to this syndrome.

## Conclusions

The BRASH syndrome represents a critical clinical entity characterized by the interplay of mild hyperkalemia and therapeutic levels of AV node blockers, particularly prevalent in the elderly population with concurrent cardiac and renal pathologies. This syndrome can lead to a dangerous cycle culminating in shock and multiple organ failure if not promptly recognized. The case presented underscores the necessity of a comprehensive assessment of patients, particularly those on multiple medications, to identify the hallmark signs of BRASH syndrome. Careful consideration of the patient's pharmacological history, along with the exclusion of alternative diagnoses, is essential to ensure timely and effective therapeutic intervention.

Furthermore, as the global population continues to age and the management of hypertension becomes increasingly stringent, the incidence of BRASH syndrome is likely to rise. This necessitates heightened awareness among healthcare professionals regarding the signs and symptoms associated with this syndrome. By understanding its underlying pathophysiology and employing prompt, aggressive treatment strategies, clinicians can significantly improve patient outcomes and mitigate the risks of morbidity and mortality associated with BRASH syndrome.

## References

[1] Bonvini RF, Hendiri T, Anwar A (2006). Sinus arrest and moderate hyperkalemia. Ann Cardiol Angeiol (Paris).

[2] Ata F, Yasir M, Javed S, Bilal ABI, Muthanna B, Minhas B, Chaudhry HS (2021). Diagnostic and therapeutic challenges of BRASH syndrome: a case report. Medicine Case Reports and Study Protocols.

[3] Farkas JD, Long B, Koyfman A, Menson K (2020). BRASH syndrome: bradycardia, renal failure, AV blockade, shock, and hyperkalemia. J Emerg Med.

[4] Hollander-Rodriguez JC, James F, Calvert J (2006). Hyperkalemia. Am Fam Phy.

[5] Aziz EF, Javed F, Korniyenko A, Pratap B, Cordova JP, Alviar CL, Herzog E (2011). Mild hyperkalemia and low eGFR a tedious recipe for cardiac disaster in the elderly: an unusual reversible cause of syncope and heart block. Heart Int.

[6] Vuckovic K, Richlin D (2004). Bradycardia induced by hyperkalemia. AAOHN J.

[7] Lee TH, Salomon DR, Rayment CM (1986). Hypotension and sinus arrest with exercise-induced hyperkalemia and combined verapamil/propranolol therapy. Am J Med.

[8] Palmer BF, Carrero JJ, Clegg DJ (2021). Clinical management of hyperkalemia. Mayo Clin Proc.

